# COVID-19 and the heart: what we have learnt so far

**DOI:** 10.1136/postgradmedj-2020-138284

**Published:** 2020-09-17

**Authors:** Kunal Bikram Shaha, Dhiraj Narayan Manandhar, Jung Rae Cho, Ashok Adhikari, Man Bahadur K C

**Affiliations:** Internal Medicine, Cardiology., Patan Academy of Health sciences, Lalitpur, Nepal; Nephrology, Nepal Medical College Teaching Hospital, Kathmandu, Nepal; Cardiology, Interventional., Kangnam Sacred Heart Hospital,HUMC, Yeongdeungpo-gu, Korea (the Republic of); Internal Medicine, Cardiology., Patan Academy of Health sciences, Lalitpur, Nepal; Cardiology, Electrophysiology, Shahid Gangalal National Heart Centre, Kathmandu, Nepal

**Keywords:** COVID-19, SARS-Cov-2, Carditis, Arrhythmias, Angiotensin receptor blocker

## Abstract

Since the outbreak of COVID-19 or coronavirus disease caused by severe acute respiratory syndrome coronavirus 2 from Wuhan, China, the cardiology fraternity’s interest has been drawn towards the pandemic with a high case fatality rate of 10.5% and 6% in patients with heart disease and hypertension, respectively. One of the postulated mechanisms for this high fatality rate is the possible abundance of ACE type 2 receptor in the cardiovascular system that strongly binds with the spike protein of COVID-19 and helps internalise into the cell resulting in acute cardiac injury (ACI). More than 7% of cases with COVID-19 are reported to have this type of ACI. A tenfold rise in mortality has been observed in patients with COVID-19 who experience a rise in high-sensitivity (hs)-troponin. All most half of the patients who died of COVID-19 had a rise in hs-troponin. More than 15% of cases with COVID-19 experienced different types of arrhythmias. All these statistics denote how important cardiovascular pathology is in patients with COVID-19. Controversies of renin–angiotensin–aldosterone system inhibitors usage in patients with COVID-19 and meticulous handling of case with acute coronary syndrome categorically stresses cardiologists to bust the myths hovering around and set a standard guideline to counterfeit the fatality with timely diagnosis and treatment of COVID-19–induced ACI. In this review, we sought to summarise the current evidence of COVID-19-associated cardiac injury and suggest the implications for its proper diagnosis and treatment.

## Introduction: Acute Cardiac Injury in Patients with Covid-19

**Figure 1:**
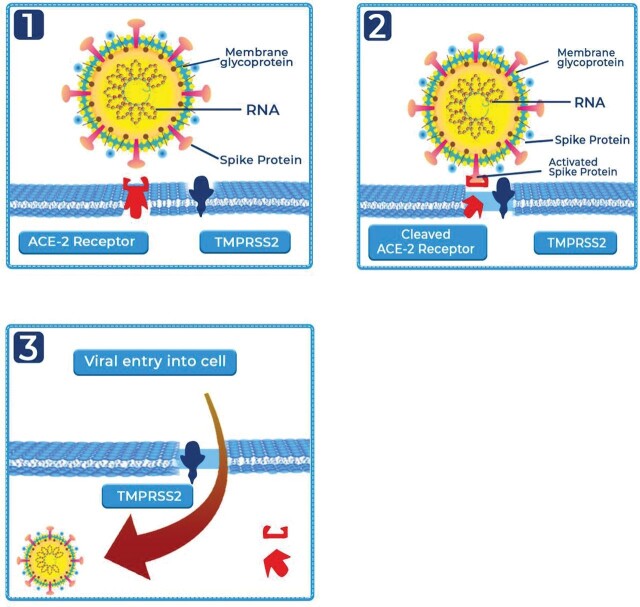
Three-step SARS-CoV-2 virus internalisation in host cell. SARS-CoV-2, severe acute respiratory syndrome coronavirus 2; TMPRSS2, type 2 transmembrane protease receptor.

Adapted from Rabi *et al.*^6^

As the lung being the prime site of pathology in patients with COVID-19, the heart scores second as a target organ of severe acute respiratory syndrome coronavirus 2 (SARS-CoV-2). The reason may be an abundance of ACE type 2 (ACE-2) receptor in the heart, which helps the virus get easily internalised into the cells.^1 2^ The scRNA-seq data from the human heart showed that more than 7.5% of myocardial cells have positive ACE-2 expression.^1^ In addition to the heart and lung, ACE-2 is expressed in the intestinal epithelium, vascular endothelium and the kidneys, providing a mechanism for the multiorgan dysfunction that can be seen with SARS-CoV-2 infection.^3 4^ ACE-2 receptor has a strong binding affinity to the surface spike protein of COVID-19 which after binding gets activated by type 2 transmembrane protease receptor and thus internalises into the host cell (figure 1).^2 5 6^

### Acute cardiac injury in patients with COVID-19

**Figure 2:**
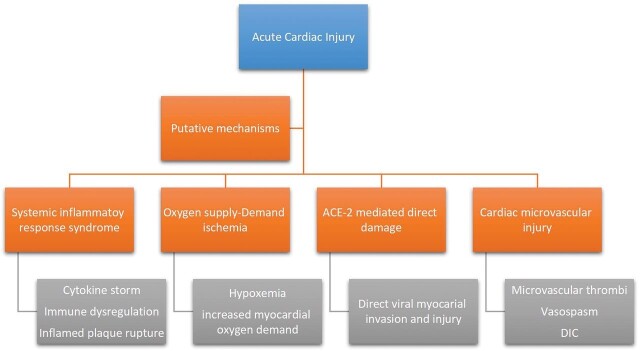
Proposed multipronged attack by COVID-19 resulting in acute cardiac injury.^6–8^ DIC, disseminated intravascular coagulation.

Various putative mechanisms (figure 2)^6–8^ of acute cardiac injury (ACI) in patients with COVID-19 have been put forward as depicted in the schema below.

### Acute COVID-19 cardiovascular syndrome^7^

- Inflammatory response in the form of systemic inflammatory response syndrome (SIRS)/cytokine storm inciting dysregulated immune response^6^ and inflamed plaque rupture leading to coronary artery thrombosis or spontaneous coronary artery dissection^8^ Culminating in acute coronary syndrome (ACS).
- Oxygen supply–demand mismatch due to hypoxia leads to ACS, especially type 2 myocardial infarction (MI).^8^
- Microvascular injury as a result of microvascular thrombi formation in continuum with disseminated intravascular coagulation or vasospasm or dysregulated immune response that surges in after viral response culminates into ACI and left ventricular (LV) dysfunction/heart failure.^8^
- Direct cardiotropic myocardial Injury^8^: SARS-CoV-2 induces cellular level damage by inducing oxidative stress and intracellular acidosis causing mitochondrial damage, hence promotes cardiac myocyte apoptosis.
- SARS-CoV-2–induced ACE-mediated damage^8^: owing to abundant ACE-2 receptor in cardiovascular disorder, florid SARS-CoV-2 internalisation is promoted culminating into severe COVID-19. SARS-CoV-2 subsequently induces hyperstimulation of the ACE-1 pathway that incites vasoconstriction, inflammation, fibrosis and proliferation promoting adverse myocardial remodelling in addition to acute lung injury. On the other hand, SARS-CoV-2 inhibits the cardioprotective ACE-2 pathway comprising angiotensin 1–7 effect in the form of antifibrotic, antiproliferative, anti-apoptotic and vasodilatory property. Ultimately, SARS-CoV-2 brings up all the substrate required for heart failure.

The manifestation of COVID-19 on the cardiovascular system represents a spectrum as depicted below.

- Acute myocarditis (including fulminant variant).
- ACS: MI type 1/2, non-ST-elevation MI (NSTEMI), unstable angina.
- Arrhythmias (supraventricular tachycardia/ventricular tachycardia/ventricular fibrillation (VF)).
- Heart failure with reduced and preserved ejection fraction (HFrEF/HFpEF), cardiogenic shock.
- Stress-induced cardiomyopathy.
- Acute pericarditis with or without tamponade.
- Thromboembolic complications: arterial thromboembolism, deep vein thrombosis, intracardiac thrombus, microvascular thrombi, pulmonary embolism, stroke.

The thrombotic complication has been noted as a part of acute COVID-19 cardiovascular syndrome (ACovCs) in patients with severe COVID-19, which results due to substantial coagulation activation. This coagulation cascade is activated by SIRS. Endothelial dysfunction and procoagulant milieu are created by cytokine release/storm induced by virus invasion.^9^ The procoagulant milieu is further exaggerated by hypoxia. Contrary to traditional belief, pulmonary vasculature thrombosis, both micro and macro in COVID-19, are mostly due to in situ pulmonary thrombosis rather than embolic phenomenon.^9^ Albeit different pathophysiology, the warranted parenteral anticoagulant (heparin) therapy as prophylaxis and therapeutic option still holds true.

As per Murphy *et al*,^10^ interleukins (ILs) have a key role to play in the pathophysiology of HFrEF and HFpEF in myocarditis.

- IL-1 signalling contributes to the pathogenesis of heart failure by inducing both systolic and diastolic dysfunction.^10^
- Systolic function is impaired through uncoupling of both L-type calcium channels and adenylyl cyclase to β/b-adrenergic receptors, resulting in desensitisation to endogenous or exogenous β/b-agonists.
- Diastolic dysfunction occurs due to impaired calcium reuptake by the sarcoplasmic reticulum through downregulation of phospholamban and sarcoplasmic reticulum calcium-adenosine triphosphatease.^10^

## Mechanism of Mi in COVID-19

ACI has been observed in 6–7% cases of patients with COVID-19.^11^

MI type 1 and type 2 have been proposed in patients with COVID-19 owing to

**Figure 3:**
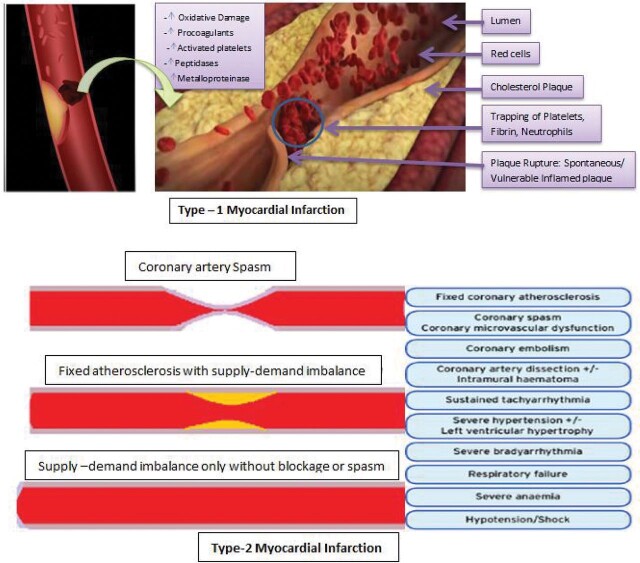
(A) Putative mechanism of type 1 MI in COVID-19. (B) Putative mechanism of type 2 MI in COVID-19. MI, myocardial infarction.

- Inflammed coronaries vulnerable to plaque rupture^12^ leading to thrombosis or spontaneous coronary artery dissection (figure 3A) and
- Demand–supply mismatch (figure 3B) in coronaries resulting from hypoxia, increased core body temperature, decreased cardiac contractility and increased heart rate,^12^ respectively.

In COVID-19, the pattern of troponin release (rise or rise and fall) in the context of a clinical presentation of type 1 or 2 MI, myocarditis or cytokine/stress-related cardiomyopathy is not well defined.^7^

The incidence of stress-related cardiomyopathy has been on rising trend during COVID-19 pandemic than the prepandemic area due to psychological, social and economic stress associated with COVID-19 pandemic rather than COVID-19 disease itself.^13^ Takotsubo syndrome is also known as stress-induced cardiomyopathy; though incompletely understood, putative mechanisms include epicardial spasm, microvascular dysfunction, direct adrenergic-receptor-mediated myocyte injury and systemic vascular effects that alter ventricular–arterial coupling. In stress-induced cardiomyopathy, the role of acute stress-induced sympathetic overactivity leading to catecholamine surge and subsequent myocardial dysfunction in the form of regional wall motion abnormality^14^ (transient apical ballooning) not following any coronary arterial territorial pattern is the rule.

ACI is characterised by marked cardiac troponin elevation accompanied by ST-segment elevation or depression on EKG, with normal epicardial coronaries.^11^ This stresses upon the fact that it is not the routine atherothrombosis rather inflammation ignited plaque rupture or coronary artery dissection that culminates into ACI as one of the putative mechanisms.

There is no standard value to differentiate acute versus chronic myocardial injury. If the first troponin level is >99th percentile, then an increase of at least 50% of the 99th percentile or a change >20% may be *considered acute*.^15 16^ A cardiac troponin result above the 99th percentile upper reference limit without a rise and/or fall over a period of serial measurements is characteristic of *chronic myocardial injury*.^17^

In accordance with Zhou *et al*,^18^ Troponin I did not elevate at the beginning of the infection rather rise was noted in patients with an increase in severity of COVID-19. Troponin I is mainly a marker of multiorgan failure and pulmonary hypertension associated with acute respiratory distress syndrome (ARDS) more than a marker that identifies patients with acute myocarditis. The elevation of troponin during hospitalisation is an important marker of prognosis and should be taken as a red flag and remain alert for severity despite normal initial level at admission.^18 19^

Hence, a rise in troponin possibly may be just a marker of hyper-inflammatory phase/cytokine storm inducing ARDS and pulmonary hypertension leading to right ventricular strain, and an important prognostic marker even in absence of ACS/myocarditis. Thus, troponin should not be only taken as a single surrogate marker for depicting ACI due to COVID-19.

## Chinese Experience with Troponin as A Diagnostic and Prognostic Tool in COVID-19

Analysis of a series of 52 critically ill patients by Yang *et al*, in China with COVID-19, revealed myocardial injury (high-sensitivity cardiac troponin I (hs-cTnI) >28 ng/L) in 29% of the patients.^20^

Analysis of a second Chinese single-centre retrospective report by Shi *et al* showed of the 416 patients hospitalised with COVID-19, approximately 20% of the patients had an acute myocardial injury (cTnI >0.04 μg/L), especially in older individual with multiple comorbidities. Even after adjusting baseline characteristics and medical comorbidities in the study, acute myocardial injury experienced higher mortality.^21^

A multicentre Chinese retrospective study by Zhou *et al*^18^ showed patients hospitalised with COVID-19,myocardial injury (cTnI >28 ng/L) was observed in 1% of survivors and 59% in non-survivors.

A meta-analysis using data from China under Lippi *et al*^22^ revealed abnormal cTnI values (>99th percentile) in 8–12% of the patients hospitalised with COVID-19, and elevations were associated with more severe disease with poor prognosis. The mean difference in cTnI value was 25.6 ng/L (95% CI 6.8 to 44.5) between those with (n=123) and without (n=218) severe disease.^18^ A tenfold rise in mortality has been observed in those with elevated hs-troponin I.

As per Shi *et al*,^21^ more than half of the patients having cardiac injury with a rise in troponin experienced in-hospital death, indicating that COVID-19–induced cardiac injury is associated with major adverse clinical outcomes. However, the exact mechanism of cardiac injury among these patients with COVID-19 remains uncertain, but as discussed earlier, various putative mechanisms have been put forward to explain the etiopathogenesis of ACI and related biomarkers.

## Cardiac Injury in Pre-Existing Cardiovascular Disease in COVID-19

ACI in patients with COVID-19 was observed more in hypertensives and those with coronary heart diseases indicating the fact that pre-existing cardiovascular disorders are more prone to ACI. Approximately 30% and 60% of the patients with cardiac injury had a history of coronary heart disease and hypertension, respectively.^21^ This implies renin–angiotensin–aldosterone system (RAAS) activation in abundance has some important role to play with SARS-CoV-2 ACE-mediated damage leading to hyperstimulation of ACE-1 pathway that incites vasoconstriction, inflammation, fibrosis and proliferation promoting adverse myocardial remodelling in addition to acute lung injury.

Elderly patients with underlying diseases are more likely to be infected with SARS-CoV-2 and tend to be severely ill, especially those with hypertension, coronary heart disease and diabetes.^23^ The prevalence of comorbidities, that is, diabetes, cardio-cerebrovascular disease (CCVD) and hypertension among patients with COVID-19 is 9.7%, 16.4% and 17.1%, respectively. Likewise, case fatality rate (CFR) of diabetes, CCVD and hypertension among patients with COVID-19 was 7.3%, 10.5%, and 6% respectively as per a meta-analysis of six published studies from China.^24 25^ This relative surge in CFR in comparison to other comorbidities emphasises the role of ACE receptor abundance in cardiovascular disorder and ACE-mediated damage by SARS-Cov-2, in the pathophysiology of patients with severe COVID-19.

## Role of NT-proBNP in ACI

Patients with severe COVID-19 who have higher N-terminal-pro-brain natriuretic peptide (NT-proBNP) levels were found to be of old age with high levels of systematic inflammatory markers. Patients with higher NT-proBNP (above 88.64 pg/mL) level had a lower cumulative survival rate. After adjusting for potential confounders in separate modes, NT-proBNP presented as an independent risk factor of in-hospital death in patients with severe COVID-19.^26^

The elevated NT-proBNP in these cases was believed owing to the cardiac complications resulted from complex interactions among pre-existing conditions, relative ischaemia, upregulation of the sympathetic system, systemic inflammation and direct pathogen-mediated damage to the cardiovascular system.^26 27^

The surprising fact is that the cut-off value of NT-proBNP(above 88.64 pg/mL) to predict the adverse outcome of severe COVID-19 has been found to be lower than the threshold, which was used to diagnose heart failure (450 pg/mL for people aged <50 years, 900 pg/mL for people aged 50–75 years and 1800 pg/mL for people aged >75 years). It was suggested that the prognostic effect of plasma NT-proBNP in patients with severe COVID-19 could not fully ascribe to heart failure induced by the virus or hypoxia.^26^

Hence, even in the absence of elevated filling pressures or clinical heart failure, one may come across a rise in NT-proBNP (lower cut-off than in heart failure) in severe COVID-19, which definitely carries a prognostic value on disease outcome besides diagnosing and prognosticating HFpEF/HFrEF in patients with COVID-19 ascribed to a higher cut-off values.

It would not be unfair to label hs-troponin and NT-proBNP as a prognostic marker of severe COVID-19 in addition to its role as a diagnostic marker in ACI.

### RAAS blockers in COVID-19

**Figure 4:**
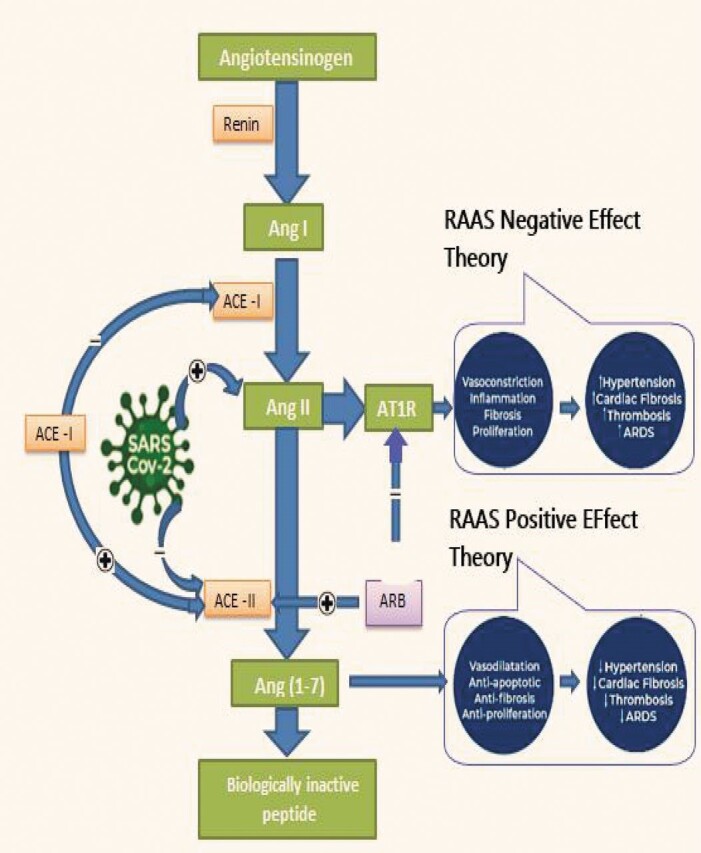
SARS-CoV-2–induced ACE-mediated damage and role of ACEI/ARB in COVID-19. ACEI, ACE inhibitor; Ang I, angiotensin I; ARB, angiotensin receptor blocker; ATIR, angiotensin II type 1 receptor.

As mentioned in the schema (figure 4), SARS CoV2 uses the ACE-2 receptor to get entry into host cell resulting into downregulation of ACE-2 receptors and hyperstimulation of ACE-1 pathway leading to vasoconstriction, inflammation, fibrosis and proliferation promoting acute lung injury and adverse myocardial remodelling.^28–30^

## Interplay of SARS-CoV-2 and RAAS; Proposed theory Against Raas Blockers use in COVID-19^28–30^

RAAS blockers: ACE inhibitors/angiotensin receptor blockers (ACEIs/ARBs) block the ACE-1 pathway and hyperstimulate the ACE-2 pathway which in turn leads to the upregulation of the ACE-2 receptors (figure 4) readily available for more internalisation of SARS-Cov-2 culminating into overwhelming severe COVID-19 as a result of the high load of viremia in host cells.

Therefore, initiation or continuation of ACE/ARBs (RAAS blockers) may promote severe COVID-19 as per this theory. Initiating other class of antihypertensive in COVID-19 should be the preferred strategy until and unless there is a serious compelling indication of ACEIs/ARBs as in acute MI or heart failure as per one of the prevailing schools of thought against the use of ACEIs/ARBs in the present context.

## Proposed Theory in Favour of Raas Blockers in COVID-19^28–30^

RAAS blockers block the ACE-1 pathway resulting into the upregulation of the ACE-2 receptors and hyperstimulation of the ACE-2 pathway (figure 4) elucidating the cardioprotective role of angiotensin 1–7 in the form of its antifibrotic, antiproliferative, anti-apoptotic and vasodilatory property (figure 4).

Hence, the initiation of ACEI/ARB may be beneficial in downregulating the ACE-1-mediated damage by SARS-CoV-2 which may be just at the cost of more internalisation of the virus by upregulating ACE-2 receptors, which is still to be proven in clinical grounds.

## Chinese Clinical Experience with Raas Blockers

Recently, Zhang *et al*^31^ studied the ‘Association of inpatient use of angiotensin-converting enzyme inhibitors and angiotensin II receptor blockers with mortality among patients with hypertension hospitalised with COVID-19’ and concluded that inpatient use of ACEIs/ARBs was associated with a lower risk of all-cause mortality compared with ACEIs/ARBs non-users. It emphasised that even in the presence of possible confounding variables, it was unlikely that in-hospital use of ACEIs/ARBs was associated with an increased mortality risk or any harm hence advocating its safety.

Li *et al*^32^ studied 362 patients with hypertension of case series of 1178 hospitalised patients with COVID in regard to the association of renin–angiotensin system inhibitors with severity or risk of death. The in-hospital mortality with hypertension was 21.3%. The percentage of patients with hypertension taking ACEIs/ARBs did not differ between those with severe and non-severe infections (32.9% vs 30.7%; p=0.645) nor did it differ between non-survivors and survivors (27.3% vs 33.0%; p=0.34). This study emphasised that ACEIs/ARBs are not associated with the severity or mortality of patients with COVID-19.

The advocacy of societal guidelines of not discontinuing ACEIs/ARBs in COVID-19 is a consensus statement rather than based on clinical trials. Albeit this fact on the clinical ground is further supported by studies by Zhang *et al*^31^ and Li *et al.*^32^

The tug of war between RAAS positive and negative effect theory has come to an end as Mancia *et al*^33^ unearthed the fact that ACEIs/ARBs did not affect the risk of contracting COVID-19. Supporting the same school of thought, Reynold *et al*^34^ revealed that none of the antihypertensive classes including ACEIs/ARBs had any positive association with SARS-Cov-2 positivity and worsening of COVID-19 disease. Though all the studies right from Zhang *et al* until Reynold *et al* are observational in nature with limitation of few possible confounders, the common truth unearthed is ‘RAAS blockers are safe in COVID-19’ further supported by the fact that these observational studies include a large number of participants from a different race, ethnicity and geography.

*Exogenous supplement of rhACE2*, the recombinant human ACE-2, helps in relieving lung injuries in several acute pneumonia experimental models and also exhibits the ability to prevent angiotensin II–induced hypertension, myocardial hypertrophy, diastolic dysfunction and myocardial fibrosis.^28^

Additionally, soluble ACE-2 can neutralise the spike protein on the surface of the SARS-CoV-2, thus inhibiting the entry of viruses into host cells. Exogenous supplement of rhACE2 may be a good way to prevent and treat COVID-19.^28^ Human trial is warranted in the future to establish its efficacy and safety.

## Arrhythmias in Patients with COVID-19

In a recent report from Wuhan by Wang *et al*,^11^ 16.7% of hospitalised and 44.4% of intensive care unit patients with COVID-19 had arrhythmias. Arrhythmia is induced by hypokalemia in COVID-19 disease; this results due to the interaction between SARS-CoV2 and the RAAS system, which is a matter of concern indeed.^35^ Alone or complex interplay of dyselectrolytemia, electrically unstable inflamed myocardium, LV dysfunction, myocardial ischaemia, hypoxia and acidosis due to acute lung injury may be the putative mechanism behind the origin of arrhythmias.

Even in some anecdotal cases of COVID-19 with late myocardial dysfunction, cardiopulmonary arrest with pulseless electrical activity or VF has been reported during the recovery phase of their pulmonary illness. Almost all forms of supraventricular and ventricular tachyarrhythmias have been documented. Although less discussion on bradyarrhythmias has been observed so far, we cannot deny its presence looking into the pathophysiology of COVID-19 on the basis of anecdotal experience.

### Drug-induced arrhythmias

QTc-prolonging drugs like chloroquine (CQ)/hydroxychloroquine (HCQ), azithromycin, lopinavir, ritonavir, which are used as trial therapeutics in COVID-19 alone or in combination, are to be used with caution owing to their Torsades de pointes generating potential.

Tisdale score, a QT prolongation predicting tool developed by Tisdale,^36^ can be quite handy regarding judicious use of QT-prolonging drug in the background of the prevailing clinical scenario. Tisdale has assigned scores 1–3 with a maximum risk score of 21 with the score assigned as per the below-mentioned parameters.

- Age ≥68 years, females and use of loop diuretics: Score 1 for each.
- Serum potassium ≥3.5 mEq/L, admission QTc ≥450 ms, patients with acute MI: Score 2 for each.
- Use of one QTc-prolonging drugs, sepsis, heart failure: Score 3 for each.

Note: Additional three points have been assigned for use of two or more QTc-prolonging drugs, which imply we get a sum making a score of 6 if the patient is taking two or more QTc-prolonging drugs.

According to the risk score assigned, the risk level has been classified as follows:

Low risk: ≤6 points

Moderate risk: 7–10 points

High risk: ≥11 points

### Suggestion: (based on anecdotal experience and scientific utility of Tisdale score)

QT-prolonging Therapeutic drug for COVID-19 is not advisable to start if any of the following is encountered.

- Congenital long QT syndrome.
- Baseline QTc >500 ms if QRS <120 ms.
- Baseline QTc >530 ms if QRS >120 ms
- Abnormal serum levels of potassium, calcium and magnesium.
- Tisdale score more than or equal to 11, that is, high risk.

After initiating any QT-prolonging therapeutic drug in COVID-19

- If the Tisdale score is in between 1 and 10 (low-–moderate risk) and QTc increases by 60 ms then risk vs benefit has to be outweighed regarding the QT-prolonging drug continuation in patients with COVID-19.
- When the Tisdale score is between 1 and 10 (low–moderate risk) and absolute QTc value is >500 ms (if QRS <120 ms) OR Absolute QTc is >550 ms (if QRS >120 ms), then risk versus benefit has to be outweighed regarding the continuation of QT-prolonging drug in patients with COVID-19.

Note: ECG to be done at baseline and 2–4 hours post first and second doses of the drug then daily until the drug is continued.

### Clinical stages of COVID-19^18^

COVID-19 can be classified primarily into three stages as depicted below:

### Stage I (Early infection)

The early infection stage comprises viral response with mild constitutional symptoms: Fever >99.6°F, dry cough, diarrhoea, headache and signs such as lymphopenia, prolonged prothrombin time, increased D-dimer and raised lactate dehydrogenase.

### Stage II (Pulmonary phase)

It indicates host inflammatory response primarily affecting lungs with symptoms such as shortness of breath and air hunger, and signs such as PaO_2_/FIO_2_ ≤300 mm Hg, abnormal chest imaging, transaminitis and low normal procalcitonin.

### Stage III (Hyperinflammation)

It is a continuum of host inflammatory response of the pulmonary phase representing cytokine dysregulation/storm. The patient may show features of ARDS, shock/SIRS, ACI and heart failure. Lab findings elucidated in this stage are a rise in inflammatory markers, which include serum, lactate dehydrogenase, IL-6, D-dimer and ACI markers like troponin, NT-proBNP.

### Clinical course in COVID-19

In a retrospective multicentre cohort study,^18^ 191 patients (135 from Jinyintan Hospital and 56 from Wuhan Pulmonary Hospital) were studied, of whom 137 were discharged and 54 died in hospital. Table 1 summarises the clinical parameters of these patients. ACI, as per Zhou *et al*,^18^ inflicts at a median of 15 days from the onset of illness in non-survivors. Death due to COVID-19 was at a median of 19 days from the onset of illness. Patients who survived were discharged at a median of 22 days from the onset of illness in survivors.

**Table 1:** Clinical course in COVID-19 survivors and non-survivors (Zhou *et al*)^18^

Clinical parameters in survivors	Period of illness (on average)	Duration (average)
Fever	Day 1–Day 12	12 days
Cough	Day 1–Day 19	19 days
Dyspnoea	Day 7–Day 19	13 days
ICU admission	Day 12–Day 18	7 days
Sytemic corticosteroid	Day 12–Day 19	8 days
SARS-CoV-2 RNA positive	Day 1–Day 20	20 days
Discharge	At Day 22	After 22 days
**Clinical parameters in non-survivors**	**Period of illness (average)**	**Duration (average)**
Fever	Day1–Day 13	13 days
Cough	Day 1–Day 16	16 days
Dyspnoea	Day7–Day19	13 days
ICU admission	Day12–Day 19	8 days
Sytemic corticosteroid	Day 13–Day 19	8 days
SARS-CoV-2 RNA positive	Day 1–Day 20	20 days
Invasive ventilation	Day 15–Day 19	5 days
Sepsis	Inflicted on Day 10	
ARDS	Inflicted on Day 12	
Acute kidney injury	Inflicted on Day 15	
Acute cardiac injury	Inflicted on Day 15	
Secondary infection	Inflicted on Day 17	
Death	Occurred at Day 19	

ARDS, acute respiratory distress syndrome; ICU, intensive care unit; SARS-CoV-2, severe acute respiratory syndrome coronavirus 2.

In this study, potential risk factors like older age, high sequential organ failure score and d-dimer >1 μg/mL were taken as a poor prognostic marker in the early stages of COVID-19.

Based on prolong viral shedding which has been recorded up to 20 days in survivors can help formulate the period required for isolation and exploration of potential therapeutics.

### Treatment and essential consideration

**Figure 5:**
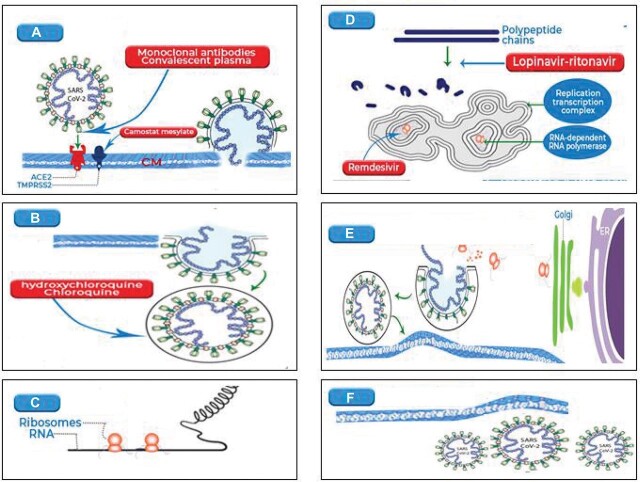
SARS-CoV-2 virology and possible therapeutic intervention. (A) Fusion. (B) Entocytosis. (C) Translation. (D) Proteolysis. (E) Translation and RNA replication. (F) Virion release. SARS-CoV-2, severe acute respiratory syndrome coronavirus 2.

At present, no drugs or vaccines are available for the treatment of COVID-19. At this time of the pandemic, a way to resourcefully control COVID-19 and the spread of pandemic is drug repositioning. Several interventional treatments, some even at experimental stages, for COVID-19 are being advocated without clear efficacy and safety considerations. The drugs that are being tried for the treatment of COVID-19 include antimalarials, antivirals, IL inhibitors, interferons, kinase inhibitors, corticosteroids^37^ (figure 5: optional), etc. We discuss a few of these drugs in this section.

A case report from China showed treatment with methylprednisolone, immunoglobulin and other supportive measures improved significantly fulminant cardiomyopathy (left ventricular ejection fraction (LVEF): 27%) with cardiogenic shock due to SARS-CoV-2 in a 37-year-old man. LVEF returned to 66% and markers of cardiac injury including NT-proBNP returned to normal values.^38^

#### CQ and HCQ

CQ or HCQ has antiviral activity. The entry of SARS-CoV into the cells is inhibited by interfering with glycosylation of its cellular ACE-2 receptor. In addition, HCQ inhibits T cell activation, expression of CD145 and cytokines release. Studies until now on HCQ alone or with azithromycin are inconclusive in reducing viral shedding and time to clinical recovery. Treatment with these agents has adverse events like hypoglycemia, QT prolongation and risk of Torsades de pointes, a potentially life-threatening arrhythmia. Hashem *et al* recommended to use CQ and HCQ rationally until high quality randomised clinical trials clarify their role in the treatment or prevention of COVID-19.^39^ After screening 45 articles, Shah S *et al* ^40^ reviewed five articles and found a lack of evidence of efficacy in preventing COVID-19 though preclinical results were promising. Three preclinical studies and two clinical opinions showed prophylactic effects of CQ and HCQ. High-quality RCTs and observational studies will prove the fact on prophylaxis with CQ or HCQ against COVID-19 as this may convey a false sense of security.

WHO,^41^ Food and Drug Administration (FDA)^42^ and National Institute of Health (NIH)^43^ all are of the same opinion that CQ and HCQ should not be used in hospitalised patients as there were safety issues and little or no reduction in the mortality of hospitalised patients with COVID-19.

#### Antiviral agents

Antivirals like ritonavir, ribavirin and lopinavir have been used globally against SARS-CoV-2. In one of the studies done in China, it was found that favipiravir was more potent in antiviral action than that of lopinavir/ritonavir without any significant adverse reactions.^44^

#### Lopinavir/ritonavir

Lopinavir (a protease inhibitor) disrupts viral replication, and ritonavir increases the half-life of lopinavir by inhibiting cytochrome P450 3A.^45^ Cao *et al* ^46^ randomised 199 hospitalised patients with COVID-19 and lopinavir/ritonavir combination did not accelerate clinical improvement or decrease mortality at Day 28 when compared to standard care alone. There were some limitations as it was open-labelled and had confounder like the use of glucocorticoid. Evidence on the efficacy and safety of lopinavir/ritonavir in patients with COVID 19 is still limited and controversial.

Chong *et al* ^47^ shared their experience on the treatment of COVID-19 in which HCQ was added in 11 patients after starting lopinavir/ritonavir. Five patients had new events of prolonged QTc after starting HCQ which normalised with discontinuation.

WHO discontinued trials on lopinavir/ritonavir as they showed little or no reduction in the mortality of hospitalised patients with COVID-19.^41^ This drug should not be used in ACovCS as the risk of arrhythmias may increase because of pre-existing cardiac structural or functional abnormalities, concomitant ventricular arrhythmias or a prolonged QT interval at baseline.^7^

#### Remdesivir

Remdesivir, a broad-spectrum antiviral drug, inhibits RNA-dependent RNA polymerase, a protein essential for viral replication. Wang *et al* ^48^ showed that remdesivir/CQ was highly effective in vitro. One of the preliminary results reported earlier time to recover of 11 days (95% CI 9 to 12), in remdesivir group (n=538) when compared with 15 days (95% CI 13 to 19) in the placebo group (n=521) and HR for death was 0.70 (95% CI 0.47 to 1.04). They concluded that remdesivir was superior in reducing the time to recovery in hospitalised patients with COVID-19 who had evidence of lower respiratory tract infection.^49^ On the contrary, a randomised trial^50^ from China did not find statistically significant clinical benefits but had a reduction in time to clinical improvement in those who were treated earlier with remdesivir. Early treatment was necessary in non-human primate models of SARS and MERS, which argues use of remdesivir earlier in patients with COVID-19. This needs confirmation with large multicentered and multiraced study.

#### Immunomodulators

Marked elevations of IL-6 and other inflammatory markers demonstrating cytokine activation may indicate the role of IL-6 inhibitors like sarilumab, siltuximab and tocilizumab in ACovCS and severe COVID-19. Thus, tocilizumab may partially rescue SARS-CoV-2-associated immune dysregulation.^51^ Tocilizumab seemed to be an effective treatment and a new platform for a therapeutic strategy for severe COVID-19. With the use of tocilizumab in patients with COVID-19, there was a normalisation of CT scan changes, reduction in inflammatory markers and need for oxygen, and reduced ventilation.^52 53^ The average duration of hospitalisation was 13.5 days with no obvious side effects observed during the study. However, there were many limitations in the study done by Alattar *et al* it being retrospective, the use of multiple interventions, and the lack of verification of serum IL-6 levels before and after tocilizumab. Preliminary reports on tocilizumab are encouraging and ongoing randomizsed controlled trials (RCT) will help clear the place of it in the treatment of COVID-19. It is recommended that before starting tocilizumab latent tuberculosis should be ruled out.

#### ACE-2 activators

The ACE-2 activators may be a possible therapeutic strategy to treat COVID-19. ACE-2 is abundantly expressed in the heart, lungs, brain, blood vessels, central nervos system and macrophages. The loss of ACE-2 enhances susceptibility to heart failure.^54 55^ Likewise, a decrease in ACE-2 brings on pneumonia and its activation decreases the inflammatory response in the lungs.^56^ When ACE-2 is activated, it will prevent binding of SARS-CoV-2 to ACE-2 and check entry into the cell and prevents fibrosis and lung injury by promoting the effect of the enzyme on different organs. Xanthenone or diminazene aceturate are some examples of ACE-2 activators that are being used in cattle to treat trypanosomiasis.^54 57^

#### Corticosteroids

In a recently conducted landmark RECOVERY trial, patients with COVID-19 were randomised to receive oral or intravenous dexamethasone and to receive standard of care to look at the 28-day mortality. Dexamethasone usage resulted in lower 28-day mortality among those who were receiving either invasive mechanical ventilation (29.3% vs 41.4%; rate ratio, 0.64; 95% CI 0.51 to 0.81) or oxygen alone (23.3% vs 26.2%; rate ratio, 0.82; 95% CI 0.72 to 0.94) but not among those receiving no respiratory support.^58^Treatment guideline of NIH^43^ recommends the use of other forms of corticosteroid if dexamethasone is not available.

#### Others

Extracorporeal membrane oxygenation (ECMO) was used in selected cases with refractory shock or ventricular arrhythmias caused by ACovCS. Case reports have described the successful rescue of patients with cardiogenic shock with the use of ECMO.^7^ In one of the case reports,^59^ a 69-year-old patient who had respiratory distress, hypotension and cardiogenic shock was rescued in 5 days with the use of ECMO. So, if the facility is available then a trial with ECMO can be done in patients who present with ACovCS and cardiogenic shock.

#### Convalescent plasma

The convalescent plasma from recovered patients with COVID-19 has been approved recently by the FDA.^60^ Shen *et al* ^61^ studied five critically ill patients with COVID-19 with convalescent plasma. The study noticed an improvement in clinical status. But being a small uncontrolled case series requires further validated randomised clinical trials to support this notion.^61^

#### Adjunctive therapy^43^:

- Antithrombotic therapy
- Vitamin C, D
- Zinc supplement

Some other drugs like ivermectin,^62^ inhibitors of glucosylceramide synthase,^63^ bevacizumab^64^ are under trial for treatment of COVID-19 and ACovCS.

## Suggestions in The Management of Patients with Cardiovascular Disease in The COVID-19 ERA

The management of patients with cardiovascular disease in the COVID-19 era has to be focused on how to diagnose as early as possible and treat the patients according to the best available strategy, while not sacrificing the safety of healthcare providers. Here are the suggestions based on the risk versus benefit approach.

- Along with appropriate cardiac management of COVID-19 confirmed and suspected cases, consideration of maximum safety of healthcare workers has to be equally emphasised and prioritised based on risk versus benefit approach on behalf of both the patients and healthcare workers.
- Unnecessary invasive and non-invasive cardiac testing (EKG, transthoracic/transesophageal/stress echocardiography, cardiac catheterisation) should be avoided until deemed necessary weighing benefit versus risk to both patients and health workers.
- Transesophageal and transthoracic echocardiography or urgent catheterisation if deemed necessary should be performed after being equipped with full personal protective equipment (PPE) in patients with COVID-19.
- In cases of ST-elevation MI (STEMI) in patients with COVID-19, thrombolysis using streptokinase/tenecteplase should be the preferred modality of treatment except in high-risk cases.
- High-risk cases of STEMI (eg, GRACE score >140) should be taken to catheterisation lab (cath lab) for primary angioplasty if cath lab is fully equipped with proper PPE and COVID-19 infection control management plan including safe transfer without/minimal potential exposure risk to healthcare workers.
- Non-STEMI cases should be taken to cath lab with full PPE, once two consecutive RT-PCR (reverse transcriptase PCR) for the COVID-19 genome is negative.
- Electrophysiological study and ablation to be performed with full PPE in cases of life-threatening arrhythmias.
- Pacemaker, implantable cardioverter defibrillator (ICD), cardiac resynchronising therapy (CRT) with or without ICD should be implanted using full PPE weighing risk versus benefit as in other cardiac emergencies.
- Considering the high prevalence of pulmonary thromboembolism in patients with severe COVID-19, adequate deep vein thrombosis prophylaxis and management of acute pulmonary embolism with anticoagulant as per standard guideline is imperative.
- Fluid restriction to be advocated in all cases with COVID-19 heart failure.
- Pneumococcal and influenza vaccines to be administered to patients with cardiovascular disease not infected with COVID-19 to prevent secondary infection in the future if one contracts COVID-19.
- Telemedicine consultation to be provided to patients with a cardiovascular disorder with minor issues or those who can be seen in the outpatient department.

**Main messages**

- Acute cardiac injury in COVID-19 and the role of troponin and NT-proBNP.
- RAAS blockers (ACEI/ARB) and arrhythmias in COVID-19.
- Treatment and essential consideration in COVID-19 era.

**Current research questions**

- How can acute cardiac injury be differentiated from multiple organ dysfunction syndrome–induced myocardial injury?
- Role and safety of RAAS blockers in COVID-19 via RCTs
- Exact mechanism of etiopathogenesis and mortality reducing therapeutics in acute cardiovascular COVID-19 syndrome.

**Key references**

- Hendren NS, Drazner MH, Bozkurt B, *et al.* Description and proposed management of the acute COVID-19 cardiovascular syndrome. doi:10.1161/CIRCULATIONAHA.120.047349.
- Yang X, Yu Y, Xu J, *et al.* Clinical course and outcomes of critically ill patients with SARS-CoV-2 pneumonia in Wuhan, China: a single-centered, retrospective, observational study. *Lancet Respir Med* 2020;8:475–81. [Erratum in: *Lancet Respir Med* 2020;8:e26]. doi:10.1016/S2213-2600(20)30079-5.
- Shi S, Qin M, Shen B, *et al.* Association of cardiac injury with mortality in hospitalized patients with COVID-19 in Wuhan, China. *JAMA Cardiol* 2020;e200950. doi:10.1001/jamacardio.2020.0950.
- Gao L, Jiang D, XS W, *et al.* Prognostic value of NT-proBNP in patients with severe COVID-19. *Respir Res* 2020;21:83.
- Guo J, Huang Z, Lin L, *et al.* Coronavirus disease 2019 (COVID-19) and cardiovascular disease: a viewpoint on the potential influence of angiotensin-converting enzyme inhibitors/angiotensin receptor blockers on onset and severity of severe acute respiratory syndrome coronavirus 2 infection*. J Am Heart Assoc* 2020;9:e016219. doi:10.1161/JAHA.120.016219.

**Self-assessment questions**

1. Acute cardiovascular COVID-19 syndrome including acute cardiac injury (ACI) is seen in COVID-19.
  True
  False
2. Acute cardiovascular COVID-19 syndrome comprises which of the following in COVID-19.
  - Acute myocarditis.
  - Acute coronary syndrome (ACS),
  - arrhythmias (SVT/VT/VF)
  - Heart failure/cardiogenic shock.
  - Stress-induced cardiomyopathy
  - Acute pericarditis with or without tamponade
  - Thromboembolic complications: arterial thromboembolism, deep vein thrombosis, intracardiac thrombus, microvascular thrombi, pulmonary embolism, stroke
  - All of the above
3. The risk of mortality increases by tenfold in patients with high hs-troponin (high-sensitivity troponin) and carries prognostic importance in COVID-19.
  True
  False
4. Tenfold rise in mortality is seen in which of the following biomarker in COVID-19.
  - High-sensitivity troponin
  - Lactate dehydrogenase
  - CKMB
  - Myoglobin
5. Even in the absence of elevated filling pressures or clinical heart failure, one may come across arise in NT-proBNP (lower cut-off than in heart failure) in severe COVID-19 which carries aprognostic value on disease outcome besides diagnosing and prognosticating heart failure in COVID-19.
  True
  False
6. The cut-off value of NT-proBNP to predict the adverse outcome of severe COVID-19 has been found to be
  - Less than that of heart failure
  - More than that of heart failure
  - Same as that of heart failure
  - Ten times more than that of heart failure
7. Large observational studies by Zhang *etal*, Li *etal*, Mancia *etal* and Reynold *etal* have a common inference that patient with COVID-19 should continue RAAS inhibitors as it is asafe co-prescription supporting the same notion endorsed by American and European Society Guidelines.
  True
  False
8. As per large observational studies by Zhang *etal*, Li *etal*, Mancia *etal*, Reynold *etal*, RAAS blockers (ACEI/ARB) in COVID-19
  - Should be discontinued
  - Should be continued
  - Increases the risk of contracting COVID-19
  - Increases severity of COVID-19
9. Ongoing various trials are underway to prove efficacy, mortality and morbidity reducing the capacity of various drugs like remdesivir, tocilizumab, HCQ, but until date, the race is still on to find the definite treatment strategy in patients with COVID-19 except few evidence of early virus clearance from the body by aforementioned drugs.
  True
  False
10. Which of the following drug has mortality reducing benefit?
  - Ivermectin
  - Tocilizumab
  - Hydroxycholoroquine
  - None of the above

**Answers**

True
(H)
True
(A)
True
(A)
True
(B)
True
(D)

## Summary

The COVID-19 has become the major health issues on the globe with its high fatality rate. Cardiovascular manifestations of ACovCs including acute cardiac injury ought not to be underestimated in these patients. An evidence-based approach incorporating early detection and management is crucial in the management of these patients with essential consideration based on risk versus benefit analysis. Being COVID-19 a novel and rapidly evolving science, more insights and updates are expected in near future, so complete coverage of all the evidence and facts is one of the limitations of this review. Inspite of this, it has attempted to summarise most of the important facts related to etiopathogenesis, clinical course, diagnosis and management of the ACovCs/ACI.

The focus of future research should try to unearth the exact etiopathogenesis of ACovCs to nail down efficacious and mortality reducing treatment. Large multicentred, multiethnic, multiraced, RCT are required to examine accurately and more precisely, the facts and evidence that we have collected so far to formulate realtime mortality reducing management.

## Supplementary Material

postgradmedj-97-655-DC1-inline-supplementary-material-1 Figure 1. Mechanism of HFrEF & HFpEFwith defined role of IL-1.

## References

[1] Zou X, Chen K, Zou J, et al. Single-cell RNA-seq data analysis on the receptor ACE2 expression reveals the potential risk of different human organs vulnerable to 2019-nCoV infection. Front Med 2020;14:185–92. 32170560 10.1007/s11684-020-0754-0 PMC7088738

[2] Wrapp D, Wang N, Corbett KS, et al. Cryo-EM structure of the 2019-nCoV spike in the perfusion conformation. Science 2020;367:1260–3. 32075877 10.1126/science.abb2507 PMC7164637

[3] Tikellis C, Thomas MC. Angiotensin-converting enzyme 2 (ACE2) is a key modulator of the renin angiotensin system in health and disease. Int J Pept 2012;2012:256294. 10.1155/2012/256294fpage 22536270 PMC3321295

[4] Zhang H, Penninger JM, Li Y, et al. Angiotensin-converting enzyme 2 (ACE2) as a SARS-CoV-2 receptor: molecular mechanisms and potential therapeutic target. Intensive Care Med 2020;46:586–90. 32125455 10.1007/s00134-020-05985-9 PMC7079879

[5] Clerkin KJ, Fried JA, Raikhelkar J, et al. COVID-19 and cardiovascular disease. Circulation 2020;141:1648–55. 32200663 10.1161/CIRCULATIONAHA.120.046941

[6] Rabi FA, Al Zoubi MS, Kasasbeh GA, et al. CoV-2 and coronavirus disease 2019: what we know so far. Pathogens 2020;9:231. 32245083 10.3390/pathogens9030231 PMC7157541

[7] Hendren NS, Drazner MH, Bozkurt B, et al. Description and proposed management of the acute COVID-19 cardiovascular syndrome. 10.1161/CIRCULATIONAHA.120.047349 PMC7314493 32297796

[8] Tersalvi G, Vicenzi M, Calabretta D, et al. Elevated troponin in patients with coronavirus disease 2019: possible mechanisms. J Card Fail 2020;26 470–5. 32315733 10.1016/j.cardfail.2020.04.009 PMC7166030

[9] Al-Ani F, Chehade S, Lazo-Langner A. Thrombosis risk associated with COVID-19 infection. A scoping review. Thromb Res 2020;192:152–60. 32485418 10.1016/j.thromres.2020.05.039 PMC7255332

[10] Murphy SP, Kakkar R, McCarthy CP, et al. Inflammation in heart failure. J Am Coll Cardiol 2020;75:1324–40. 32192660 10.1016/j.jacc.2020.01.014

[11] Wang D, Hu B, Hu C, et al. Clinical characteristics of 138 hospitalized patients with 2019 novel coronavirus-infected pneumonia in Wuhan, China JAMA 2020;323:1061–9. 32031570 10.1001/jama.2020.1585 PMC7042881

[12] Musher MD, Abers MS, Corrales VF, et al. Acute infection and myocardial infarction. N Engl J Med 2019;380:171–6. 30625066 10.1056/NEJMra1808137

[13] Jabri A, Kalra A, Kumar A, et al. Incidence of stress cardiomyopathy during the coronavirus disease 2019 pandemic. JAMA Netw Open 2020;3:e2014780. 10.1001/jamanetworkopen.2020.14780 PMC7348683 32644140

[14] Wittstein IS . The sympathetic nervous system in the pathogenesis of Takotsubo syndrome. Heart Fail Clin 2016;12:485–98. 27638019 10.1016/j.hfc.2016.06.012

[15] Inciardi RM, Lupi L, Zaccone G, et al. Cardiac involvement in a patient with coronavirus disease 2019 (COVID-19). JAMA Cardiol 2020;5:1–6. 32219357 10.1001/jamacardio.2020.1096 PMC7364333

[16] Wu AHB, Christenson RH, Greene DN, et al. Clinical laboratory practice recommendations for the use of cardiac troponin in acute coronary syndrome: expert opinion from the academy of the American Association for Clinical Chemistry and the Task Force on Clinical Applications of Cardiac Bio-markers of the International Federation of Clinical Chemistry and Laboratory Medicine. ClinChem 2018;64:645–55. 10.1373/clinchem.2017.277186 29343532

[17] American College of Cardiology, McCarthy CP . Potential implications related to diagnosing type 2 MI versus myocardial injury. Available https://www.acc.org/latest-in-cardiology/articles/2020/02/14/08/49/potential-implications-related-to-diagnosing-type-2-mi-versus-myocardial-injury (accessed 28th Mar 2020)

[18] Zhou F, Yu T, Du R, et al. Clinical course and risk factors for mortality of adult inpatients with COVID-19 in Wuhan, China: a retrospective cohort study. Lancet 2020;395:1054–62. [Erratum in: *Lancet* 2020;395(10229):1038]. 32171076 10.1016/S0140-6736(20)30566-3 PMC7270627

[19] Ammirati E, Wang DW SARS-CoV-2 inflames the heart. The importance of awareness of myocardial injury in COVID-19 patients. Int J Cardiol 2020;311:122–3. 32276774 10.1016/j.ijcard.2020.03.086 PMC7134214

[20] Yang X, Yu Y, Xu J, et al. Clinical course and outcomes of critically ill patients with SARS-CoV-2 pneumonia in Wuhan, China: a single-centered, retrospective, observational study. Lancet Respir Med 2020;8:475–81. [Erratum in: *Lancet Respir Med* 2020;8:e26]. 32105632 10.1016/S2213-2600(20)30079-5 PMC7102538

[21] Shi S, Qin M, Shen B, et al. Association of cardiac injury with mortality in hospitalized patients with COVID-19 in Wuhan, China. JAMA Cardiol 2020;e200950. 10.1001/jamacardio.2020.0950 PMC7097841 32211816

[22] Lippi G, Lavie CJ, Sanchis-Gomar F. Cardiac troponin I in patients with coronavirus disease 2019 (COVID-19): evidence from a meta-analysis. Prog Cardiovasc Dis 2020;63:390–1. 32169400 10.1016/j.pcad.2020.03.001 PMC7127395

[23] National Health Commission of People’s Republic of China . Diagnosis and treatment of pneumonia caused by novel coronavirus (trial version 4). (In Chinese). 2020. Available

[24] Wu Z, McGoogan JM. Characteristics of and important lessons from the coronavirus disease 2019 (COVID-19) outbreak in China: summary of a report of 72 314 cases from the Chinese Center for Disease Control and Prevention. JAMA 2020;323:1239. 32091533 10.1001/jama.2020.2648

[25] Li B, Yang J, Zhao F, et al. Prevalence and impact of cardiovascular metabolic diseases on COVID-19 in China. Clin Res Cardiol 2020;109:531–8. 32161990 10.1007/s00392-020-01626-9 PMC7087935

[26] Gao L, Jiang D, Wen XS, et al. Prognostic value of NT-proBNP in patients with severe COVID-19. Respir Res 2020;21:83. 32293449 10.1186/s12931-020-01352-w PMC7156898

[27] Restrepo MI, Reyes LF. Pneumonia as a cardiovascular disease. Respirology 2018;23:250–9. 29325222 10.1111/resp.13233

[28] Guo J, Huang Z, Lin L, et al. Coronavirus disease 2019 (COVID-19) and cardiovascular disease: a viewpoint on the potential influence of angiotensin-converting enzyme inhibitors/angiotensin receptor blockers on onset and severity of severe acute respiratory syndrome coronavirus 2 infection. J Am Heart Assoc 2020;9:e016219. 10.1161/JAHA.120.016219 PMC7428639 32233755

[29] Kuba K, Imai Y, Rao S, et al. A crucial role of angiotensin converting enzyme 2 (ACE2) in SARS coronavirus-induced lung injury. Nat Med 2005;11:875–9. 16007097 10.1038/nm1267 PMC7095783

[30] Vaduganathan M, Vardeny O, Michel T, et al. Renin-angiotensin-aldosterone system inhibitors in patients with COVID-19. N Engl J Med 2020;382:1653–9. 32227760 10.1056/NEJMsr2005760 PMC7121452

[31] Zhang P, Zhu L, Cai J, et al. Association of inpatient use of angiotensin-converting enzyme inhibitors and angiotensin II receptor blockers with mortality among patients with hypertension hospitalized with COVID-19. Circ Res 2020;126:1671–81. 32302265 10.1161/CIRCRESAHA.120.317134 PMC7265882

[32] Li J, Wang X, Chen J, et al. Association of renin-angiotensin system inhibitors with severity or risk of death in patients with hypertension hospitalized for coronavirus disease 2019 (COVID-19) infection in Wuhan, China. JAMA Cardiol 2020;5:1–6. 32324209 10.1001/jamacardio.2020.1624 PMC7180726

[33] Mancia G, Rea F, Ludergnani M, et al. Renin-angiotensin-aldosterone system blockers and the risk of COVID-19. N Engl J Med 2020;382:2431–40. 32356627 10.1056/NEJMoa2006923 PMC7206933

[34] Reynolds HR, Adhikari S, Pulgarin C, et al. Renin-angiotensin-aldosterone system inhibitors and risk of COVID-19. N Engl J Med 2020;382:2441–8. 32356628 10.1056/NEJMoa2008975 PMC7206932

[35] Xiong TY, Redwood S, Prendergast B, et al. Coronaviruses and the cardiovascular system: acute and long-term implications. Eur Heart J 2020:1798–1800. 10.1093/eurheartj/ehaa231 PMC7454513 32186331

[36] Tisdale JE, Jaynes HA, Kingery JR, et al. Development and validation of a risk score to predict QT interval prolongation in hospitalized patients. Circ Cardiovasc Qual Outcomes 2013;6:479–87. [Erratum in: *Circ Cardiovasc Qual Outcomes* 2013;6:e57]. 23716032 10.1161/CIRCOUTCOMES.113.000152 PMC3788679

[37] Sharma P, Tripathi S, Patel SK, et al. SARS-CoV-2/COVID-19 and its transmission, prevention, treatment and control: an update. J Pure Appl Microbiol 2020;14:945–56.

[38] Hu H, Ma F, Wei X, et al. Coronavirus fulminant myocarditis saved with glucocorticoid and human immunoglobulin. Eur Heart J 2020;ehaa190. 10.1093/eurheartj/ehaa190 PMC7184348 32176300

[39] Hashem AM, Alghamdi BS, Algaissi AA, et al. Therapeutic use of chloroquine and hydroxychloroquine in COVID-19 and other viral infections: a narrative review. Travel Med Infect Dis 2020;35:101735. 32387694 10.1016/j.tmaid.2020.101735 PMC7202851

[40] Shah S, Das S, Jain A, et al. A systematic review of the prophylactic role of chloroquine and hydroxychloroquine in coronavirus disease-19 (COVID-19). Int J Rheum Dis 2020;23:613–9. 32281213 10.1111/1756-185X.13842 PMC7262257

[41] World Health Organization . WHO discontinues hydroxychloroquine and lopinavir/ritonavir treatment arms for COVID-19 . Available https://www.who.int/news-room/detail/04-07-2020-who-discontinues-hydroxychloroquine-and-lopinavir-ritonavir-treatment-arms-for-COVID-19 (accessed 2 Aug 2020)

[42] Food US, Administration D. FDA cautions against use of hydroxychloroquine or chloroquine for COVID-19 outside of the hospital setting or a clinical trial due to risk of heart rhythm problems. Available https://www.fda.gov/drugs/drug-safety-and-availability/fda-cautions-against-use-hydroxychloroquine-or-chloroquine-COVID-19-outside-hospital-setting-or (accessed 2 Aug 2020)

[43] National Institutes of Health . Coronavirus disease 2019 (COVID-19) treatment guidelines. Available https://www.COVID-19treatmentguidelines.nih.gov/ (accessed 2 Aug 2020) 34003615

[44] Dong L, Hu S, Gao J Discovering drugs to treat coronavirus disease 2019 (COVID-19). Drug Discov Ther 2020;14:58–60. 32147628 10.5582/ddt.2020.01012

[45] The Centre for Evidence-Based Medicine . Lopinavir/ritonavir: a rapid review of effectiveness in COVID-19. Available https://www.cebm.net/COVID-19/lopinavir-ritonavir-a-rapid-review-of-the-evidence-for-effectiveness-in-treating-covid/ (accessed 2 Aug 2020)

[46] Cao B, Wang Y, Wen D, et al. A trial of lopinavir-ritonavir in adults hospitalized with severe COVID-19. N Engl J Med 2020;382:1787–99. 32187464 10.1056/NEJMoa2001282 PMC7121492

[47] Chong VH, Chong PL, Metussin D, et al. Conduction abnormalities in hydroxychloroquine add on therapy to lopinavir/ritonavir in COVID-19 J Med Virol 2020. 10.1002/jmv.26004 PMC7272857 32401368

[48] Wang M, Cao R, Zhang L, et al. Remdesivir and chloroquine effectively inhibit the recently emerged novel coronavirus (2019-nCoV) in vitro. Cell Res 2020;30;269–71. 32020029 10.1038/s41422-020-0282-0 PMC7054408

[49] Beigel JH, Tomashek KM, Dodd LE, et al. Remdesivir for the treatment of COVID-19: preliminary report N Engl J Med. 2020; 10.1056/NEJMc2022236 32649078

[50] Wang Y, Zhang D, Du G, et al. Remdesivir in adults with severe COVID-19: a randomised, double-blind, placebo-controlled, multicentre trial. Lancet 2020;395:1569–78. [Erratum in: *Lancet* 2020;395:1694]. 32423584 10.1016/S0140-6736(20)31022-9 PMC7190303

[51] Giamarellos-Bourboulis EJ, Netea MG, Rovina N, et al. Complex immune dysregulation in COVID-19 patients with severe respiratory failure. Cell Host Microbe 2020;27:992–1000. 32320677 10.1016/j.chom.2020.04.009 PMC7172841

[52] Xu X, Han M, Li T, et al. Effective treatment of severe COVID-19 patients with tocilizumab. ChinaXiv 2020. 10.1073/pnas.2005615117 PMC7245089 32350134

[53] Alattar R, Ibrahim TBH, Shaar SH, et al. Tocilizumab for the treatment of severe coronavirus disease 2019 J Med Virol 2020. 10.1002/jmv.25964 PMC7267594 32369191

[54] Rodríguez-Puertas R ACE2 activators for the treatment of COVID 19 patients. J Med Virol 2020. 10.1002/jmv.25992 PMC7267413 32379346

[55] Patel VB, Zhong JC, Grant MB, et al. Role of the ACE2/Angiotensin 1–7 axis of the renin-angiotensin system in heart failure.Circ Res 2016;118: 1313–26. 27081112 10.1161/CIRCRESAHA.116.307708 PMC4939482

[56] Fang Y, Gao F, Liu Z Angiotensin converting enzyme 2 attenuates inflammatory response and oxidative stress in hyperoxic lung injury by regulating NFκB and Nrf2 pathways. QJM 2019; 112: 914–24. 31393582 10.1093/qjmed/hcz206

[57] da Silva Oliveira GL, de Freitas RM Diminazene aceturate: an antiparasitic drug of antiquity: advances in pharmacology & therapeutics. Pharmacol Res 2015;102:138–57. 26470648 10.1016/j.phrs.2015.10.005

[58] Collaborative Group Recovery, Horby P, Lim WS, et al. Dexamethasone in hospitalized patients with COVID-19 - preliminary report. N Engl J Med 2020;

[59] Tavazzi G, Pellegrini C, Maurelli M, et al. Myocardial localization of coronavirus in COVID-19 cardiogenic shock. Eur J Heart Fail 2020;22:911–5. 32275347 10.1002/ejhf.1828 PMC7262276

[60] US Food and Drug Administration . Recommendations for investigational COVID-19 convalescent plasma. Updated 13 April 2020. Available https://www.fda.gov/vaccines-bloodbiologics/investigational-new-drug-Ind-or-device-exemption-ide-process-cber/investigationalcovid-19-convalescent-plasma-emergency-inds (accessed 28 Mar 2020)

[61] Shen C, Wang Z, Zhao F, et al. Treatment of 5 critically Ill patients with COVID-19 with convalescent plasma. JAMA 2020;323:1582–9. 32219428 10.1001/jama.2020.4783 PMC7101507

[62] Caly L, Druce JD, Catton MG, et al. The FDA-approved drug ivermectin inhibits the replication of SARS-CoV-2 in vitro. Antiviral Res. 2020;178:104787. 32251768 10.1016/j.antiviral.2020.104787 PMC7129059

[63] Vitner EB, Avraham R, Achdout H, et al. Antiviral activity of glucosylceramide synthase inhibitors against SARS-CoV-2 and other RNA virus infections. bioRxiv 2020.

[64] Scavone C, Brusco S, Bertini M, et al. Current pharmacological treatments for COVID-19: what’s next? Br J Pharmacol 2020. 10.1111/bph.15072 PMC7264618 32329520

