# Supplementary material for: COVID-19 and the heart: what we have learnt so far
Source: Postgrad Med J. 2020 Sep 17;97(1152):655–66. doi: 10.1136/postgradmedj-2020-138284 (PMC10016902; doi:10.1136/postgradmedj-2020-138284)
Supplement: postgradmedj-97-655-DC1-inline-supplementary-material-1 — Figure 1. Mechanism of HFrEF & HFpEFwith defined role of IL-1. [file postgradmedj-97-655-dc1-inline-supplementary-material-1.pdf]

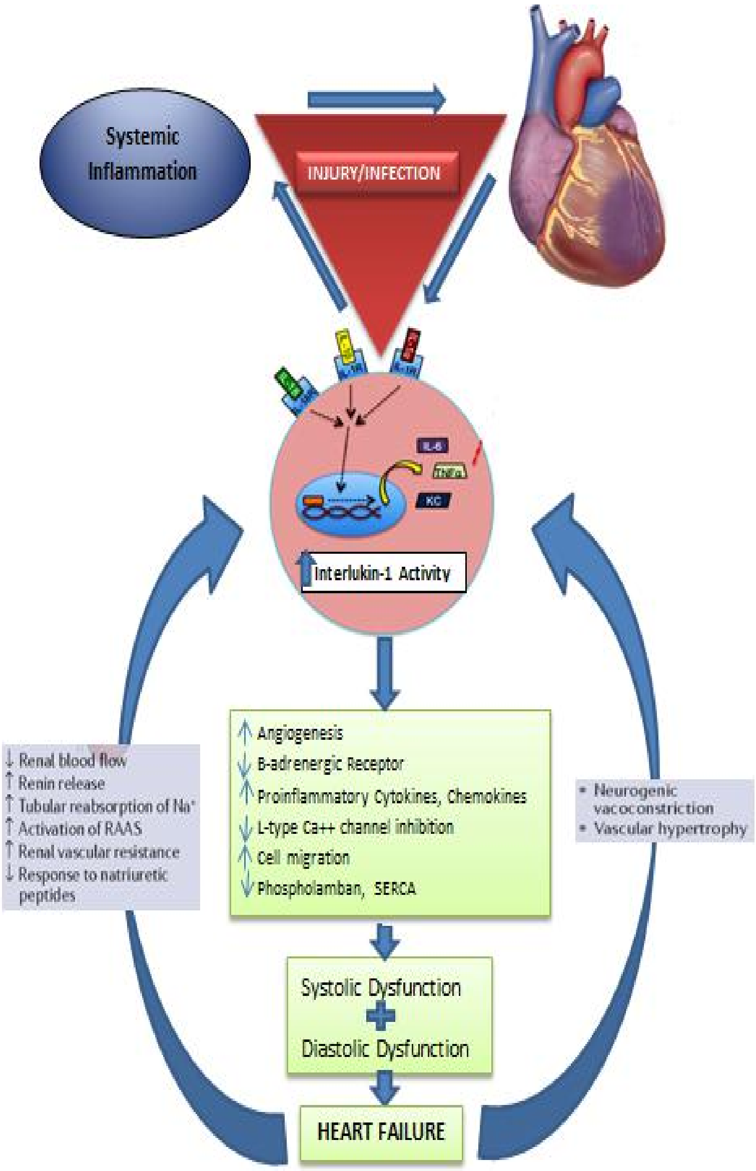

Figure 1. Mechanism of HFrEF & HFpEFwith defined role of IL-1.

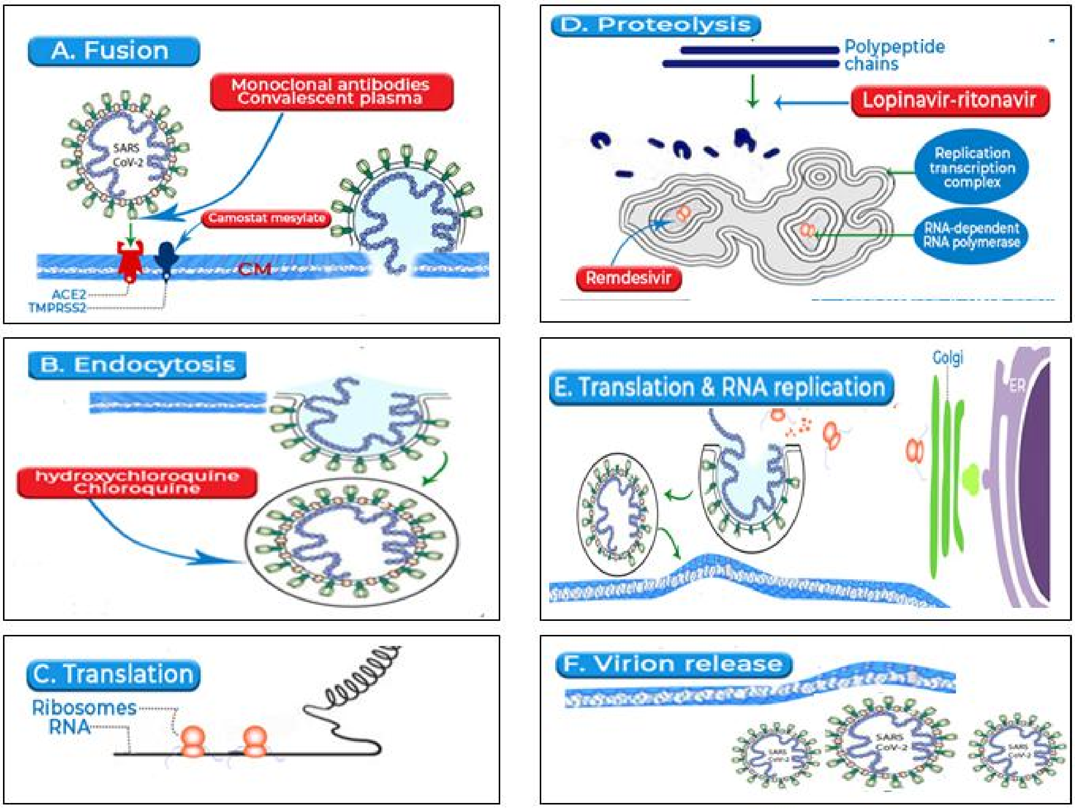

Figure 2:SARS-Cov-2 virology and possible therapeutic intervention.
